# Exogenous Melatonin Alleviates Alkaline Stress in *Malus hupehensis* Rehd. by Regulating the Biosynthesis of Polyamines

**DOI:** 10.3390/molecules22091542

**Published:** 2017-09-13

**Authors:** Xiaoqing Gong, Shuting Shi, Fangfang Dou, Yi Song, Fengwang Ma

**Affiliations:** State Key Laboratory of Crop Stress Biology for Arid Areas, College of Horticulture, Northwest A & F University, Yangling 712100, Shaanxi, China; gongxq0103@nwsuaf.edu.cn (X.G.); shuting_shi@sina.com (S.S.); a17749122448@163.com (F.D.); songyi199402@126.com (Y.S.)

**Keywords:** *Malus hupehensis* Rehd., melatonin, alkaline stress, root system architecture, antioxidant enzymes, polyamines

## Abstract

Since melatonin was identified in plants decades ago, much attention has been devoted to discovering its role in plant science. There is still a great deal to learn about the functional importance of melatonin, as well as its functional mode. In this paper, we examine the role of melatonin treatment in the response of *Malus hupehensis* Rehd. to alkaline conditions. Stressed seedlings showed chlorosis and suppressed growth. However, this phenotype was ameliorated when 5 µM melatonin was added to the irrigation solution. This supplementation was also associated with a reduction in cell membrane damage and maintenance of a normal root system architecture. Fewer reactive oxygen species (ROS) were accumulated due to the enhanced scavenging activity of antioxidant enzymes superoxide dismutase, peroxidase, and catalase. In addition, alkaline-stressed seedlings that received the melatonin supplement accumulated more polyamines compared with untreated seedlings. Transcript levels of six genes involved in polyamine synthesis, including *SAMDC1*, -*3*, and -*4*, and *SPDS1*, -*3*, and -*5*, -*6*, were upregulated in response to melatonin application. All of these results demonstrate that melatonin has a positive function in plant tolerance to alkaline stress because it regulates enzyme activity and the biosynthesis of polyamines.

## 1. Introduction

Plants are frequently threatened by a variety of biotic and abiotic stresses during their life cycle. Salt-alkaline soils are widely distributed across arid and semi-arid regions of the world, and have detrimental effects on plant growth and development [1,2]. Although soil alkalization is often associated with soil salinity, the former is considered much more hazardous to plants. This condition is generally linked with high pH stress and sodium toxicity caused by an excess of Na_2_CO_3_ and NaHCO_3_ in the soil, as well as osmotic stress. The comprehensive stress caused by alkaline soils directly affects physiological homeostasis at the cellular and whole-plant levels [3,4,5]. How plants respond to salt stress has been widely studied; for example, the SOS (Salt Overly Sensitive) pathway has been identified as one of the most important signaling pathways established in plants [6,7]. The vacuole-bound NHX-like Na^+^/H^+^ antiporters also contribute to the plant response to salt stress [8]. In contrast, less attention has been given to the mechanism by which plants respond to alkaline stress.

Plants develop various physiological and biochemical strategies to cope with stresses. Reactive oxygen species (ROS) are produced as a byproduct of several cell processes and can act as positive modulators similar to the role of phytohormones [9,10,11]. However, negative environmental stimuli can lead to excess ROS accumulation and irreversible damage, e.g., degradation of chlorophyll, lipid peroxidation, and deterioration of nucleic acids [12,13]. Although the plant response to stresses can involve different physiological and molecular changes, the accumulation of ROS is a common reaction. Therefore, plants mobilize sophisticated antioxidant systems (both enzymatic and non-enzymatic) to maintain tight control of ROS homeostasis [14,15]. For example, superoxide dismutase (SOD) is the only enzyme in plant cells that can convert excess superoxide anion (O_2_^−^) into hydrogen peroxide (H_2_O_2_), while both catalase (CAT) and peroxidase III (POD) function to convert H_2_O_2_ into water and oxygen [14,16].

Scavenging of excess ROS is a direct strategy by which plants adapt to adverse environments. However, plants can also alter their metabolism and accumulate beneficial metabolites, including polyamines (PAs). These small, flexible, nitrogen-containing compounds are found in almost all living cells, with the most common PAs in higher plants being putrescine (Put), spermidine (Spd), and spermine (Spm) [17]. Changes in polyamine (PA) metabolism are positively correlated with plant tolerance to stresses such as drought, salinity, cold, and pathogens, as well as modifications in the expression of PA biosynthetic genes [18]. For example, FcWRKY70 positively regulated Put synthesis to confer drought tolerance in *Fortunella crassifolia* [19]. Spd promotes biomass accumulation and upregulates proteins involved in cell rescue and defense as well as antioxidant enzymes in tomato (*Lycopersicon esculentum*) seedlings under high-temperature stress [20]. Exogenous Spm was proved to induce defense response and increase resistance to root rot pathogen *Phytophthora capsici* in *Capsicum annuum* plants [21]. Furthermore, a study in *Oryza sativa* L. demonstrated that Spd is vital for the adjustment of intracellular PA pathways and endogenous PA homeostasis that promotes salt tolerance [22].

Melatonin, a small, flexible, and highly conserved molecule with an indole structure, is ubiquitous in a wide range of cells, from bacterial to mammalian [23]. Since it was first reported in plants in 1995, melatonin has been shown to regulate fundamental cellular and physiological processes, e.g., stress responses [24,25]. In many plant species, melatonin confers enhanced tolerance during their responses to environmental stimuli such as salt, drought, extreme temperatures, heavy metals, or UV damage [26,27,28,29,30]. The beneficial role of melatonin in sodic alkaline stress response has also been mentioned in tomatoes [31,32]. However, whether melatonin contributes to alkaline stress in perennial woody plants remains unclear. This molecule is thought to have direct and indirect antioxidant activity [33,34,35]. Proteomics analysis of *Cucumis sativus* has indicated that melatonin facilitates ATP production as an energy source for seedlings exposed to high salinity [36]. Plants of *Medicago sativa* that are pre-treated with melatonin show better regulation of proline metabolism and nitro-oxidative homeostasis, which improves their degree of drought tolerance [28]. Under stress conditions, melatonin also strengthens several metabolic pathways, such as polyamine metabolism, ribosome pathway, amino acid metabolism, and so on [37].

As described here, we utilized a hydroponics system to simulate alkaline conditions and investigated whether exogenous melatonin could enhance stress tolerance in seedlings of *Malus hupehensis* Rehd. In particular, we focused on the potential antioxidative properties of melatonin, and examined its roles in PA metabolism and the expression of genes involved in PA synthesis.

## 2. Results

### 2.1. The Effect of Melatonin on Phenotypes of Malus hupehensis Rehd. under Alkaline Stress

**Figure 1:**
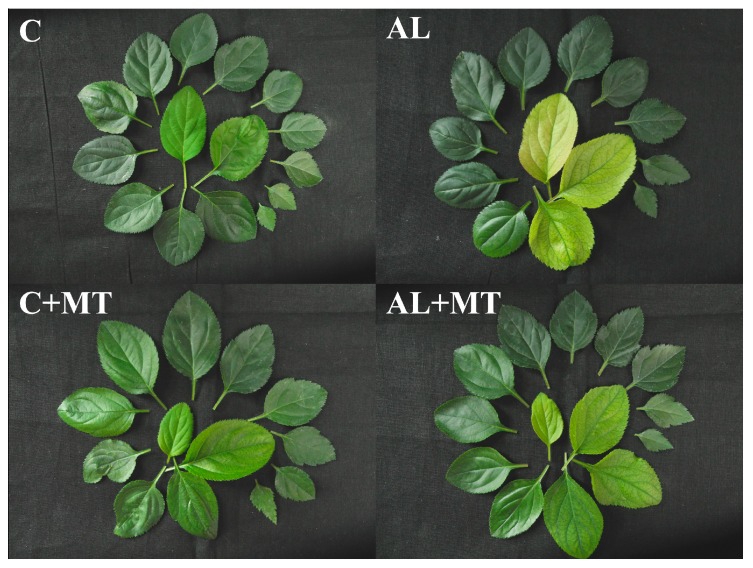
Phenotypes of *Malus hupehensis* Rehd. leaves in response to alkaline stress and/or melatonin treatment. C: normal growing conditions, pH 6.0–6.5, no melatonin treatment; AL: alkaline stress treatment with 2 mM Na_2_CO_3_/NaHCO_3_ (1:1), pH 8.5–8.8; MT: 5 µM melatonin treatment. Leaves were listed clockwise from youngest to oldest.

To determine whether supplemental melatonin can alleviate the adverse effects of alkaline stress, we added 2 mM Na_2_CO_3_/NaHCO_3_ (pH 8.5–8.8) to the hydroponics system used for growing seedlings of *Malus hupehensis* Redh. Under normal conditions (pH 6.0–6.5), the melatonin treatment had little influence on seedling morphology (Figure 1, C versus C + MT). However, when alkaline stress was induced for 15 d, young leaves from seedlings that had not received the melatonin treatment (AL group) showed obvious yellowing, while those from seedlings exposed earlier to melatonin were only slightly yellowed (Figure 1, AL versus AL + MT).

In addition, melatonin retarded the decrease in growth rates for stressed plants during the treatment period. When compared with measurements recorded on Day 0, values for shoot height, leaf numbers, and stem diameter on Day 15 were 10.7%, 14.3%, and 1.6% higher, respectively, for the AL group, and 20.9%, 30.0%, and 13.3% higher, respectively, for the C group (Table 1). Performance did not differ among the C, C + MT, and AL + MT groups. Contrasts in biomass production also indicated that the melatonin treatment was effective in easing the adverse effect of alkaline stress. The total-seedling fresh weight rose by 13.3% for the AL group, while those in the AL + MT group showed an even greater increase, i.e., 44.4% (Table 2). When dry weight was considered, values for seedlings in the AL group increased by only 11.7% versus 38.5% for those in the AL + MT group (Table 3).

### 2.2. The Effect of Melatonin on Physiological State of Malus hupehensis Rehd. under Alkaline Stress

**Figure 2:**
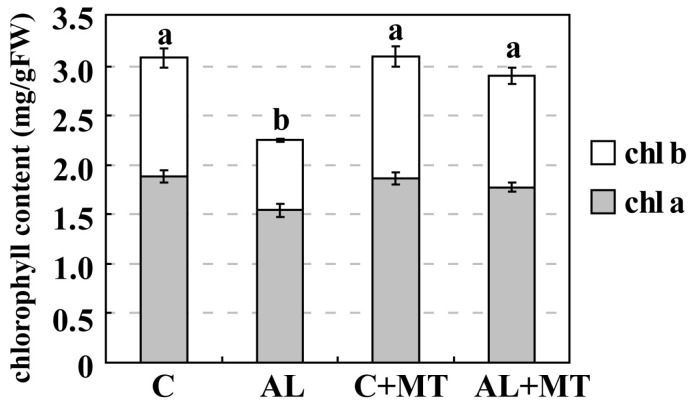
Chlorophyll (chl) concentrations in *Malus hupehensis* Rehd. seedlings in response to alkaline stress and/or melatonin treatment. Lower-case letters indicate significant differences among treatments, based on Duncan’s multiple range test (*p* < 0.05).

**Figure 3:**
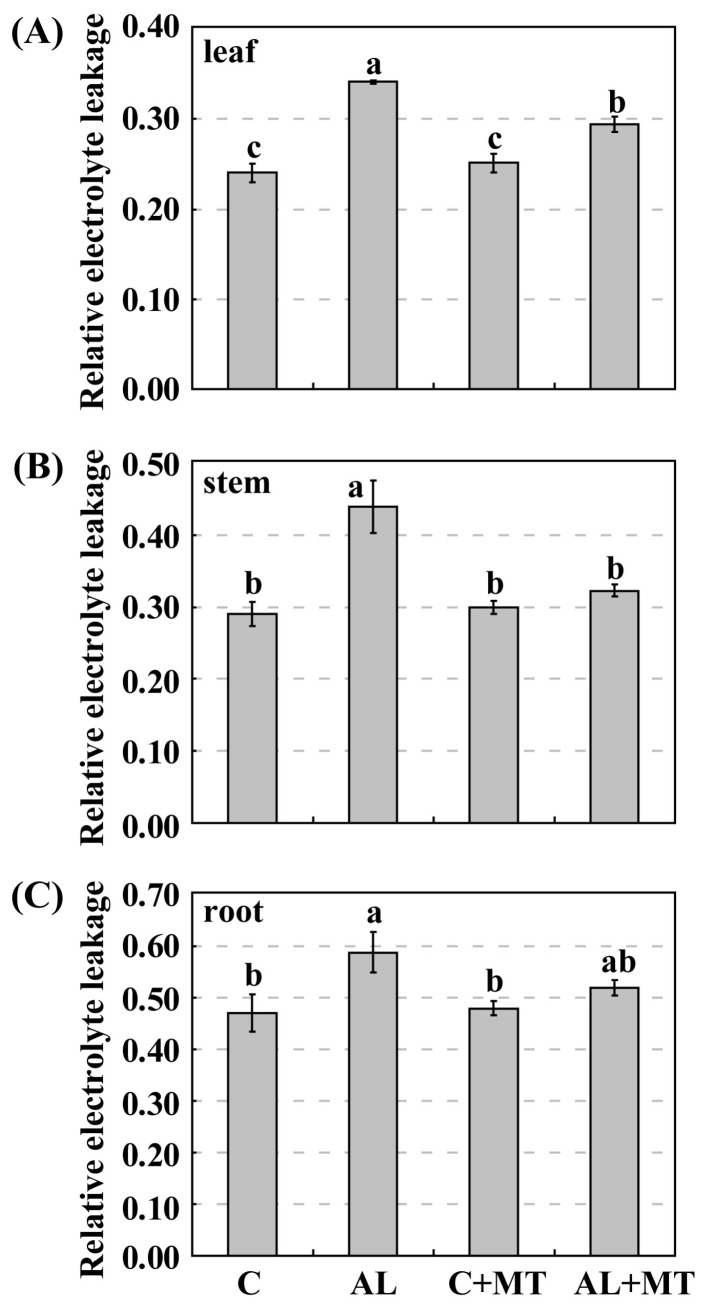
Effects of alkaline stress and melatonin treatment on relative electrolyte leakage (REL) in leaves (**A**), stems (**B**), and roots (**C**) from *Malus hupehensis* Rehd. seedlings. Lower-case letters indicate significant differences among treatments, based on Duncan’s multiple range test (*p* < 0.05).

Leaves from plants that had been treated with melatonin showed less yellowing after exposure to alkaline stress, which suggested that chlorophyll degradation had been inhibited. On Day 15, the concentrations of both chlorophyll a and b were much lower in the AL group (1.54 and 0.71 mg/g FW, respectively) (FW, fresh weight) than in the C group (1.88 and 1.20 mg/g FW, respectively), while concentrations were almost the same for the C and AL + MT groups (1.78 and 1.23 mg/g FW, respectively) (Figure 2). Values for relative electrolyte leakage (REL) at the end of the stress period were much higher in the AL group than in the C group, while REL was obviously reduced in tissues sampled from seedlings that had been treated with melatonin (Figure 3).

**Figure 4:**
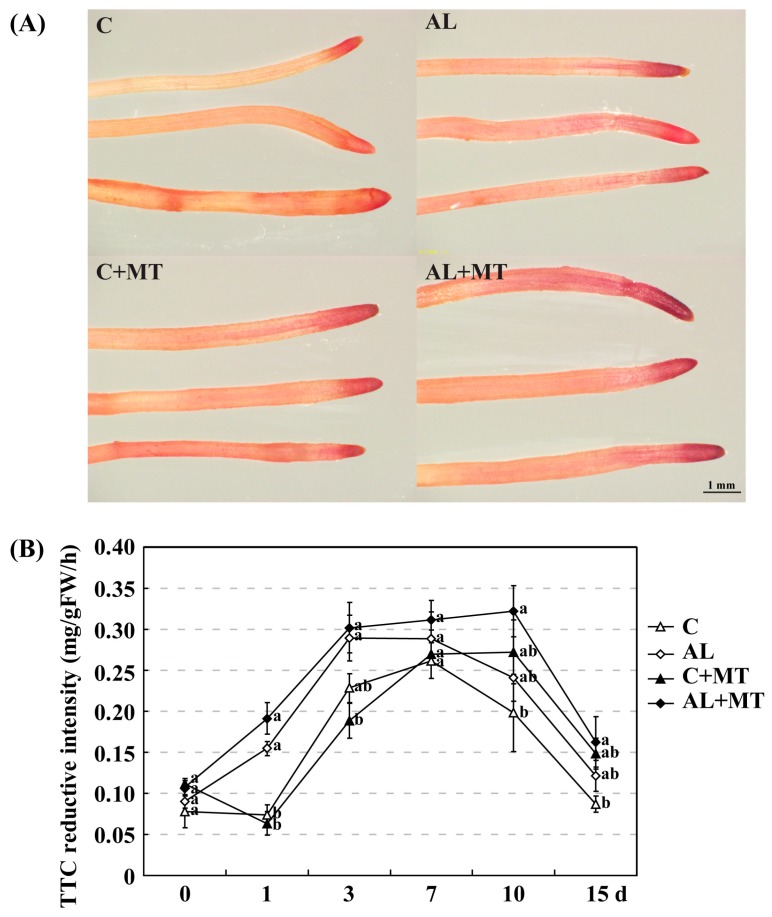
Changes in root activity for *Malus hupehensis* Rehd. seedlings exposed to alkaline stress, with or without melatonin treatment. (**A**) Absorbing roots were stained with triphenyl tetrazolium chloride (TTC) at the end of the treatment period; (**B**) TTC reductive intensity detected in roots. Lower-case letters indicate significant differences among treatments, based on Duncan’s multiple range test (*p* < 0.05).

Alkaline stress causes damage to the roots before affecting the entire plant. In AL group, root growth was also suppressed, as indicated by average length (220.46 cm), surface area (27.13 cm^2^), average diameter (0.38 mm), volume (0.29 mm^3^), and numbers of tips (1179) and forks (2002), all of which were moderately lower than the values recorded for roots in the C group (244.30 cm, 31.63 cm^2^, 0.44 mm, 0.36 mm^3^, 1577, and 2275, respectively). Treatment with melatonin promoted root growth; values obtained for those six indices were higher for the AL + MT group than for the AL group, almost equal to or even greater than those for the C group (Table 4). As a REDOX compound, triphenyl tetrazolium chloride (TTC) is commonly used as the receptor of H^+^ when analyzing the activity of different enzymes. After 15 days of alkaline stress, the root tips from seedlings in the AL and AL + MT groups were darker red than those from the C and C + MT groups. Furthermore, the color of the root tips were darker red for the C + MT and AL + MT groups than for the C and AL groups (Figure 4A). Consisted with the observed darker red in root tips from stressed AL group, the detected TTC reductive intensity was also improved when compared with the non-stressed C group, and melatonin significantly increased the TTC reductive intensity in stressed plants (Figure 4B).

### 2.3. The Effect of Melatonin on Antioxidant System in Malus hupehensis Rehd. under Alkaline Stress

**Figure 5:**
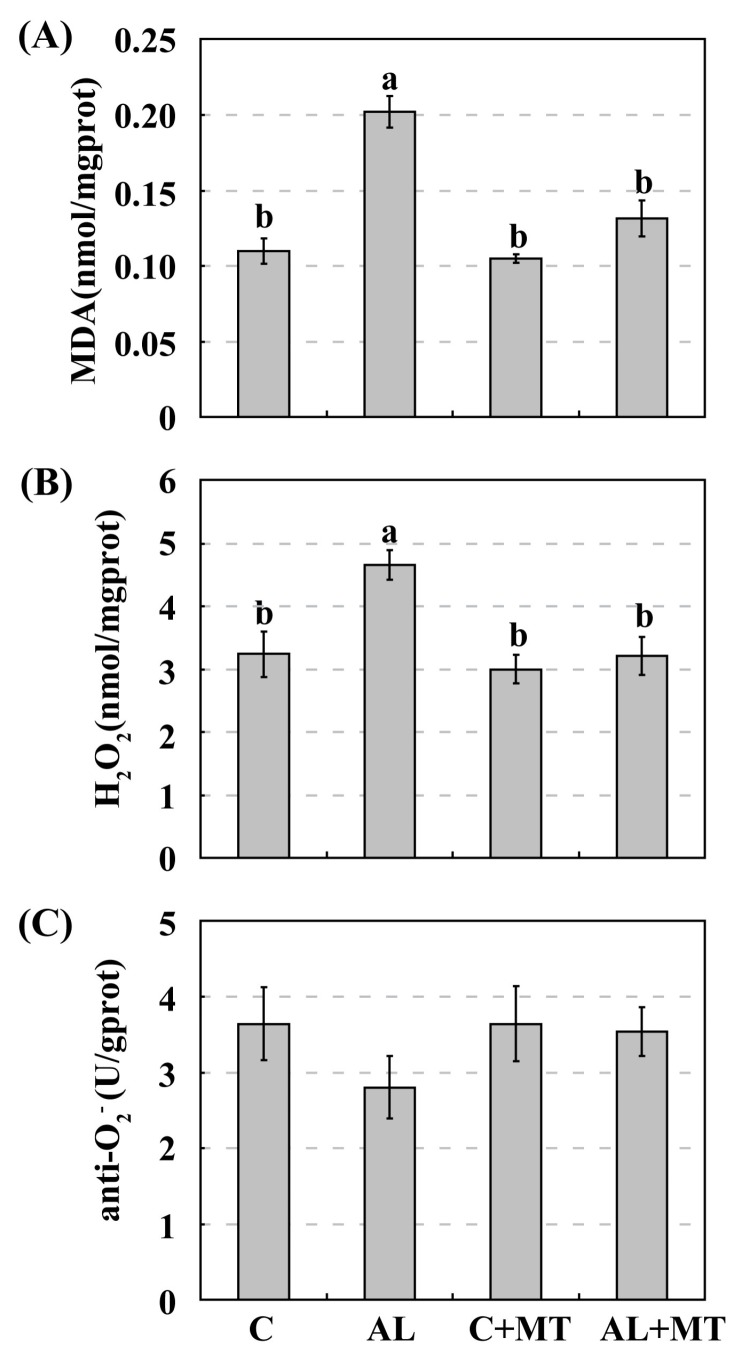
Accumulations of malondialdehyde (MDA) (**A**), hydrogen peroxide (H_2_O_2_) (**B**), and superoxide anion (O_2_^−^) (**C**) in *Malus hupehensis* Rehd. seedlings exposed to alkaline stress, with or without melatonin treatment. The accumulation of O_2_^−^ was indicated by anti- O_2_^−^ activity, which was negatively correlated with the accumulation of O_2_^−^. Lower-case letters indicate significant differences among treatments, based on Duncan’s multiple range test (*p* < 0.05).

Adverse environmental stimuli usually disrupt the cellular homeostasis of ROS, leading to oxidative stress [14]. To test our hypothesis that supplemental melatonin could cause plants to accumulate less ROS when under alkaline stress, we measured malondialdehyde (MDA) and calculated the relative levels of H_2_O_2_ and O_2_^−^. Concentrations of MDA were significantly higher in the AL group than in the other three groups, suggesting that this soil status led to severe oxidative stress in the cell membranes. Pretreated seedlings accumulated much less MDA when they were later subjected to alkaline stress (0.20 for AL versus 0.13 nmol/mg prot for AL + MT) (prot, protein), and that concentration was nearly the same as that measured in the C + MT group (0.10 nmol/mg prot) (Figure 5A). These results indicated that melatonin effectively reduced oxidative stress in the membranes under alkaline conditions. Levels of H_2_O_2_ were also significantly higher in the AL group than that in the AL + MT group (4.66 versus 3.21 nmol/mg prot), while differences were not distinct among the C, C + MT, and AL + MT groups (Figure 5B). Monitoring the activity of anti-O_2_^−^, an indicator of the O_2_^−^ level, revealed that alkaline-stressed seedlings accumulated more of that ROS, but differences in responses were not significant among the four groups (Figure 5C). This suggested that the addition of melatonin restricted the accumulation of ROS, especially H_2_O_2_, under alkaline stress.

**Figure 6:**
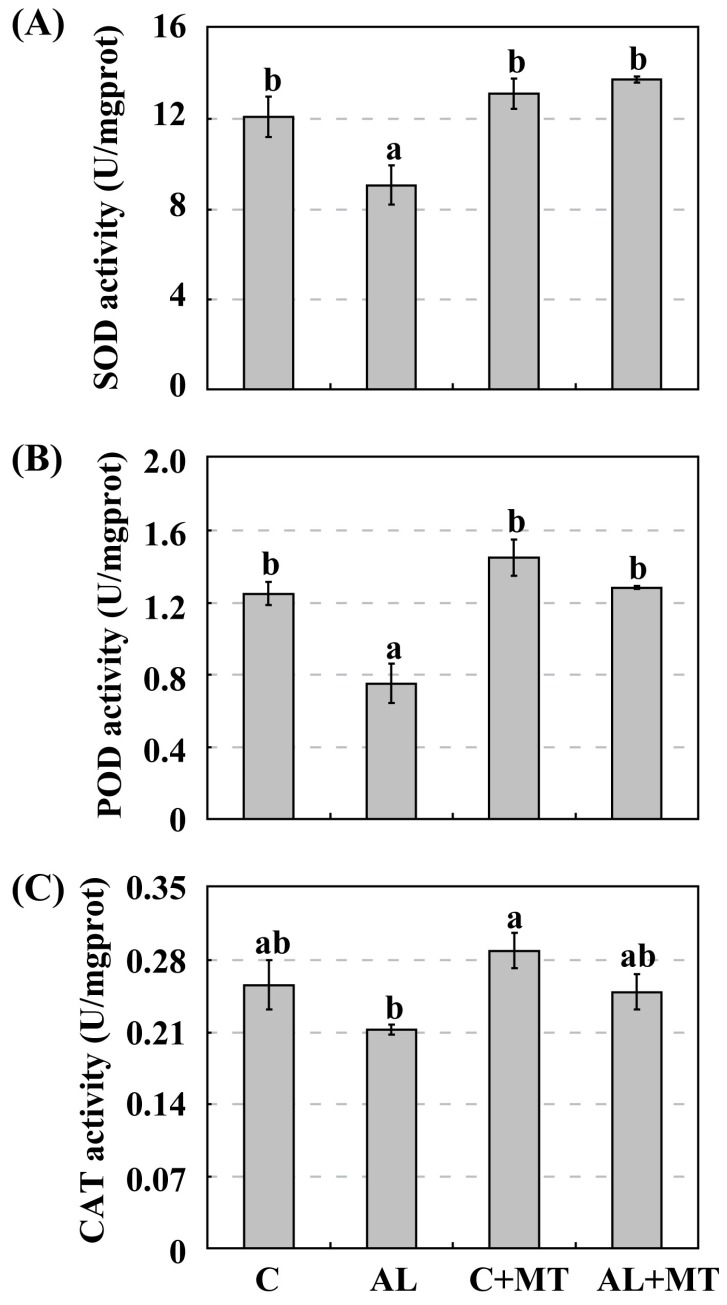
Activities of antioxidant enzymes in *Malus hupehensis* Rehd. seedlings in response to alkaline stress and/or melatonin treatment. (**A**) Superoxide dismutase (SOD); (**B**) Peroxidase III (POD); (**C**) Catalase (CAT). Lower-case letters indicate significant differences among treatments, based on Duncan’s multiple range test (*p* < 0.05).

Under normal, unstressed conditions, SOD activity was slightly higher in pretreated seedlings (13.13 U/mg prot for C + MT versus 12.10 U/mg prot for C). When alkaline stress was induced, this gap in activity widened, i.e., to 13.76 U/mg prot for AL + MT seedlings versus only 9.06 U/mg prot for the AL seedlings (Figure 6A). The same results were obtained when monitoring POD and CAT, both of which were slight strongly activated in the C + MT group than in the C group. Their activities were also much higher in the AL + MT than in the AL group (Figure 6B,C). This demonstrated that melatonin promoted the activity of antioxidant enzymes, hence scavenging the excess ROS that accumulated in plant cells under alkaline stress.

### 2.4. The Effect of Melatonin on Polyamines Biosynthesis in Malus hupehensis Rehd. under Alkaline Stress

**Figure 7:**
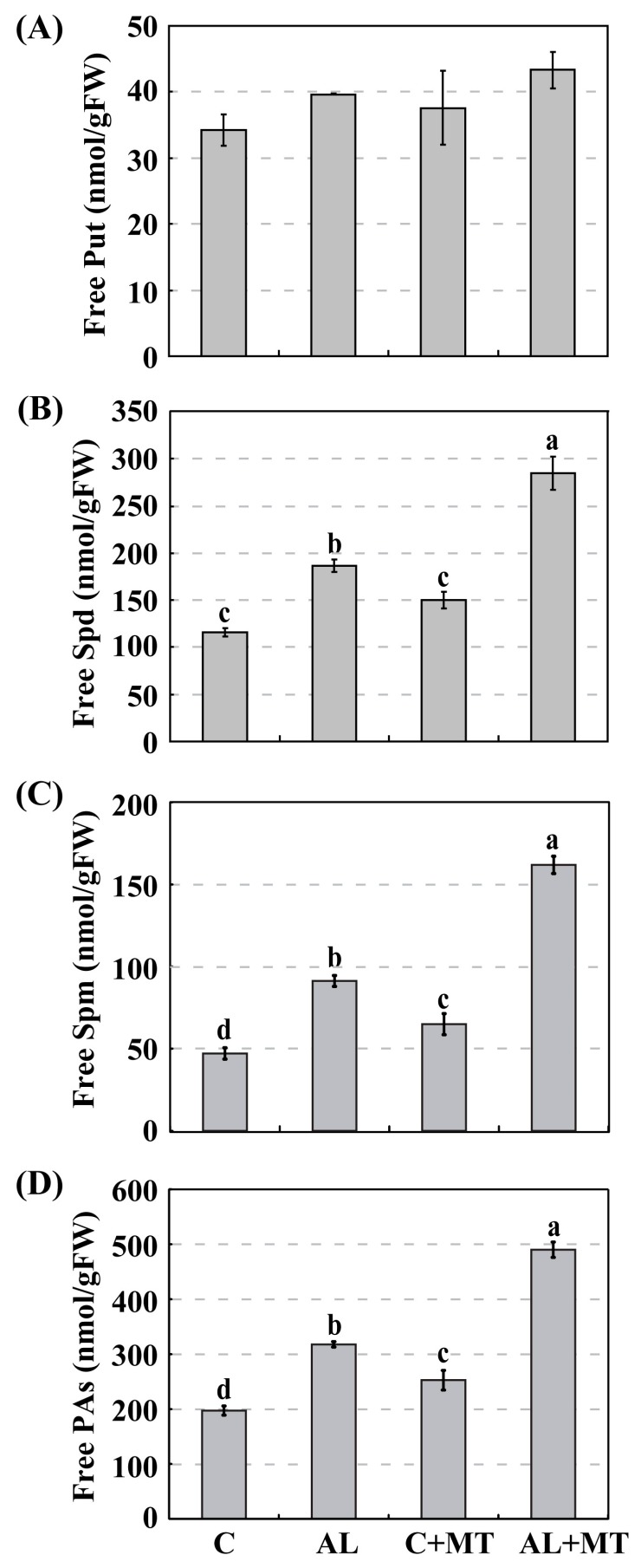
Accumulations of free polyamines (PAs) in *Malus hupehensis* Rehd. seedlings in response to alkaline stress and/or melatonin treatment. (**A**) Putrescine (Put); (**B**) Spermidine (Spd); (**C**) Spermine (Spm); (**D**) Accumulation of total free PAs. Lower-case letters indicate significant differences among treatments, based on Duncan’s multiple range test (*p* < 0.05).

Polyamines are a group of aliphatic polycations that are ubiquitously distributed in higher plants. Changes in their metabolism are positively correlated with enhanced stress tolerance [18]. To analyze whether the application of melatonin alters PA synthesis, we measured their accumulations in our treatment groups and found much higher PA accumulations in AL seedlings than in C seedlings (317.8 versus 197.3 nmol/g FW) (Figure 7). Supplemental melatonin increased those accumulations, such that PA levels were significantly higher (490.0 nmol/g FW, or approximately 1.5-fold higher) when seedlings were later exposed to alkaline stress (Figure 7D). A little more Put was accumulated in AL + MT seedlings than in those from the other three treatment groups (Figure 7A). Likewise, levels of Spd were obviously increased in response to alkaline conditions, and melatonin treatment induced this accumulation, especially in stressed seedlings (Figure 7B). A similar pattern was noted with Spm (Figure 7C). All of these results indicated that melatonin promoted the synthesis of PAs, especially Spd and Spm, under alkaline stress.

**Figure 8:**
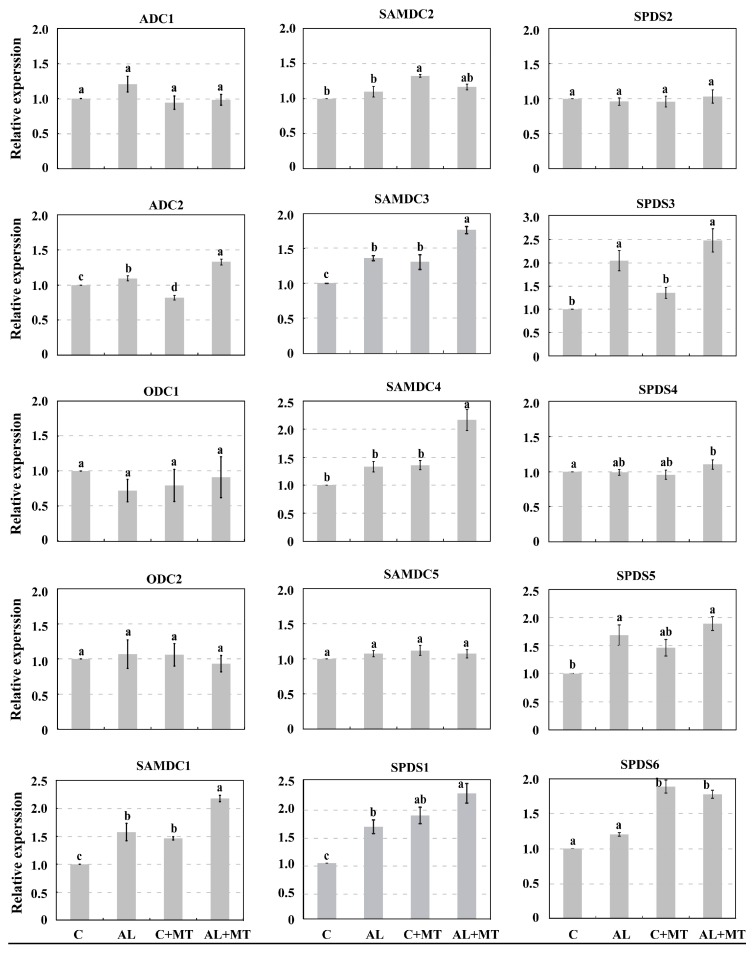
Expression of genes involved in biosynthesis of polyamines in *Malus hupehensis* Rehd. seedlings in response to alkaline stress and/or melatonin treatment. Expression levels were calculated relative to expression of *MDH*. All data are means ± SD of four replicates, and lower-case a and b indicate significant differences among treatments, based on Duncan’s multiple range test (*p* < 0.05).

We used qPCR analysis to examine the expression of genes involved in PA synthesis. They included 15 genes that were first identified from the GDR database (https://www.rosaceae.org/), and then designated according to their homologous genes in *Arabidopsis* (Supplementary Materials, Table S1). As shown in Figure 8, expressions of *ADC1*, *ODC1*, and *ODC2* were only negligibly changed by stress conditions, and the expression of *ADC2* was induced by melatonin under alkaline stress. Whereas *SAMDC5* showed only small changes in expression in response to either AL or MT treatments, *SAMDC2* was obviously induced by MT treatment and the induction disappeared under alkaline stress. *SAMDC1*, *SAMDC3*, and *SAMDC4* were induced by alkaline stress and their expression was much higher in seedlings that had received the melatonin treatment. Furthermore, expression of *SPDS1* was much higher in the AL + MT seedlings than in the other treatments. *SPDS3* and *SPDS6* were induced by alkaline stress, and expression levels of them were much higher in AL + MT seedlings comparing with that in AL seedlings. The expression profile of *SPDS5* in four groups was similar to *SPDS3*, but it was not that distinct. Finally, no obvious changes in the expression of *SPDS2* and *SPDS4* were noted among treatment groups. These results indicated that melatonin strengthened the accumulation of PAs under alkaline stress by regulating the expression of genes involved in their synthesis.

## 3. Discussion

Soil alkalization is a major abiotic stress that impairs plant growth and productivity worldwide. Saline-alkaline soils cover approximately 30% of the world’s land; in northeastern and northwestern China, these conditions continue to expand, posing a serious threat to agriculture [38,39]. Soil alkalization is coupled with soil salinity. Although many pathways have been elucidated that regulate plant responses to salt stress, fewer studies have focused on the mechanisms by which plants, especially perennial fruit trees, adapt to alkaline conditions. Our results indicated that exogenous melatonin enhances tolerance to alkaline stress in *Malus hupehensis* Rehd. by regulating the biosynthesis of polyamines.

The foliage was chlorotic and growth was retarded in response to alkaline stress. After 15 days of treatment, those seedlings were also shorter than the control and had produced very little new biomass. In *Arabidopsis* plants, endogenous melatonin levels are induced by heat stress and exogenous melatonin improves their thermotolerance [40]. When the sheep serotonin *N*-acetyltransferase gene *NAT* is expressed in rice (*Oryza sativa* cv. Dongjin), those plants produce more melatonin when compared with the wild type, and the transgenics also exhibit resistance to herbicide-induced oxidative stress [41]. Supplementing *Hordeum vulgare* plants with exogenous melatonin causes them to accumulate more ABA and enhances their tolerance to cold stress [27]. Melatonin also confers salt tolerance in watermelon (*Citrullus lanatus* L.), *Malus hupehensis* Rehd., and *Helianthus annuus* [26,42,43]. The negative effects of alkaline stress are somewhat similar to those of salt stress. We found here that, under alkaline stress, *M. hupehensis* seedlings that had been pre-treated with exogenous melatonin grew taller and were stronger than those that had not received that supplement. They were not as chlorotic, based on their higher chlorophyll concentrations, and showed less damage to their cell membranes, as evidenced by their relatively lower REL values. All of these findings, as well as those reported earlier, demonstrated that melatonin is a positive regulator of plant stress responses, and that exogenous applications improve plant tolerance to alkaline stress.

Plants require a healthy root system for water absorption and nutrient uptake. In responding to a changing soil environment, plants can modify various aspects of their root system architecture (RSA), including root length, branching, and total surface area [44,45]. When the supply of water and nitrates fluctuates, plants of *Arabidopsis* show a change in the lengths of their primary roots and basal root growth [46]. In rice, DEEPER ROOTING 1 (DRO1) is a quantitative trait locus that controls root angle. Its expression in that crop leads to deep rooting and helps plants avoid the negative effects of drought [47]. Exposing rice seedlings to a saline-alkaline medium results in significant reductions in the number of adventitious roots, total root length, and root surface area [48]. Under normal growing conditions, plants of *Lotus japonicus* display a dichotomous RSA pattern that becomes a herringbone pattern in response to alkaline stress [49]. For our seedlings, the values recorded for all six RSA parameters—root length, surface area, average diameter, volume, and numbers of tips and forks—were the lowest under stress conditions. In contrast, treatment with melatonin promoted the development of a strong RSA by modifying specific physiological processes in the root cells, and increased root activity in stressed seedlings.

As a versatile antioxidant, melatonin acts to remove excess free radicals, such as ROS and reactive nitrogen species (RNS), from cells when plants are exposed to adverse environments [34,50,51]. We also noted that the induction of alkaline stress was associated with higher production of H_2_O_2_ and O_2_^−^ when compared with the control group, and that lipid peroxidation and membrane damage were more severe under conditions with high pH. However, the application of melatonin reduced MDA concentrations and the accumulation of ROS when stress conditions were introduced, thereby preserving membrane integrity. During the removal of ROS, an antioxidant is converted to an oxidized form that has less scavenging activity. However, melatonin can be regenerated and, similarly, its metabolites can efficiently react with free radicals. Moreover, melatonin boosts the action of other antioxidant systems to remove excess free radicals [52,53,54]. This has been demonstrated in experiments with *Malus* sp., where melatonin directly scavenges H_2_O_2_ and enhances the activities of CAT and another antioxidant enzyme, ascorbate peroxidase, to detoxify H_2_O_2_ in plants under drought stress [33]. In watermelon, local application of melatonin activates those enzymes to remove excess ROS and make plants tolerant to cold stress [55]. Our monitoring of enzyme activity showed that SOD and POD were inhibited under alkaline stress, but melatonin treatment caused those activities to return to nearly the same level as detected under normal conditions. The detected CAT activity was also inhibited moderately by alkaline stress, and melatonin slightly elevated CAT activity. Although alkaline soils can be the source of numerous physiological challenges for plants, melatonin is still an effective broad-spectrum antioxidant that confers stress tolerance to *Malus* seedlings.

The accumulation of PAs is an integral part of the metabolic strategy by which plants survive under abiotic stresses. This is achieved through either exogenous applications of PAs or the generation of transgenic plants with altered expression of PA-related genes [56,57]. Increased PA levels enable plants to respond both spatially and temporally in order to avoid and overcome the effects of stress [19]. In alkaline-stressed tomato seedlings, Put is dramatically accumulated [58]. However, we did not find any significant difference in Put accumulations between the normal and stress treatments. Nevertheless, accumulations of Spd and Spm were much higher under the latter experimental condition, implying that the role of PAs in stress responses is both conserved and unique among species. We found it interesting that exogenous melatonin improved the levels of Spd and Spm in stressed seedlings, which suggested the possibility of direct or indirect cross-talk between melatonin and PAs when determining the degree of stress tolerance.

Melatonin might also have a positive role in the PA signaling pathway. For example, exogenous melatonin can significantly increase the accumulation of nitric oxide (NO) to increase plant tolerance to an iron deficiency. However, NO production is inhibited in PA-deficient plants that are sensitive to a lack of iron, which suggests that the positive function of melatonin in plants under Fe-deficient conditions depends upon PA-induced NO production [59]. The NO signaling pathway is considered a downstream signal in the melatonin signaling pathway [60]. Meanwhile, these PAs have also been shown to interact with NO in alkaline-stressed plants [58], implying indirect relation between PAs and melatonin in plants under alkaline stress. In the present study, we found that the contents of PAs were elevated by melatonin application under alkaline stress. Besides, melatonin up-regulated the expression of several genes involved in PA biosynthesis. For melatonin treated seedlings, expressions of *SAMDC1/3/4* and *SPDS1/3/5/6*, which are mainly involved in spermidine and spermine biosynthesis, were induced under alkaline stress, leading to increased accumulations of those particular PA, suggesting direct relationship between PAs and melatonin in plants under alkaline stress. Such direct evidence has also been confirmed in plants under low temperature stress. For example, cold-induced apoptosis was inhibited by exogenous melatonin in carrot suspension cells, and notable increases in Put and Spd levels were observed in melatonin-treated cells, which inhibited DNA laddering under cold stress [61]. In cucumber seedlings, melatonin mediated chilling stress tolerance was also been proved partly via the regulation of PAs metabolism. Melatonin increased the content of Put, Spd, and stabilized Spm under chilling stress [62]. When *Prunus persica* fruits treated with melatonin in cold storage, the expression of *PpADC* and *PpODC* were elevated, causing an increase in PAs contents [63]. Therefore, it is speculated that PAs metabolism might directly functions in melatonin signaling pathway to confer tolerance to alkaline stress. PAs may function indirectly in the melatonin signaling pathway through NO. Melatonin, polyamine and NO, they may role in the same network under alkaline stress, which calls for further study.

In summary, we have shown that exogenous melatonin mitigates the adverse effects that alkaline stress can have on the growth of *Malus hupehensis* seedlings. Treatment with this molecule alleviates damage to cell membranes, reducing lipid peroxidation and ROS accumulations when plants are later exposed to alkaline conditions. Melatonin positively alters RSA, elevates the activity of antioxidant enzymes, and increases levels of polyamines by enhancing the expression of genes involved in their biosynthesis. Further investigations should focus on the mechanism that regulates the relationship between melatonin and PAs.

## 4. Materials and Methods

### 4.1. Plant Materials, Melatonin Applications, and Alkaline Stress Treatment

*Malus hupehensis* Rehd., a triploid plant species typical of apomixis in that genus, shows high consistency in its development. We cultivated seedlings as described by Li et al. [26]. Briefly, seeds were cold-stratified for 50 days in winter. After germination, the seedlings were planted in plastic pots and grown for two months in a greenhouse before treatments began. To simulate alkaline stress, we utilized a hydroponics system [26]. For this, plastic basins (35 cm × 25 cm × 10 cm), each containing 5 L of ½-stength Hoagland’s nutrient solution, were painted black to protect the roots from light exposure, and covered with a foam board to support the seedlings. The hydroponics system was continuously supplied with oxygen, using an air pump, and the solution was refreshed every 3 days. During the two-week pre-culture period, the pH of the solution was adjusted between 6.0 and 6.5 with sodium hydroxide pellets or 85% phosphoric acid. 5 µM melatonin was preliminary added to the nutrient solution one week before stress, thereby creating the MT group. Melatonin was refreshed every three days along with the nutrient solution. Na_2_CO_3_/NaHCO_3_ (1:1) was applied separately to the seedlings for control and alkaline stress, designated as the C group (0 mM Na_2_CO_3_/NaHCO_3_) and AL group (2 mM Na_2_CO_3_/NaHCO_3_). The alkaline stress induction achieved a pH between 8.5 and 8.8, which is similar to that of alkaline orchard soils found in northwestern China. In all, four different treatment groups were created: C, C + MT, AL, and AL + MT. Each group contained 150 seedlings (30 × 5 replicates). The stress period spanned 15 days, and samples of leaves, stems were collected at 0 and 15 days during the period, while roots were collected at 0, 1, 3, 7, 10, and 15 days during that period. All the samples were collected from 5 replicates separately with identical numbers in each replicates.

### 4.2. Assessment of Growth Parameters

To evaluate the effects of melatonin treatment and eventual alkaline stress on seedling growth, we used several parameters as indices of performance: leaf number, shoot height, stem diameter, and fresh and dry weights of leaves, stems, and roots. For each treatment group, 20 seedlings were examined. Morphological characteristics were analyzed for roots harvested at the end of the treatment period, and samples were visualized with an Epson digital scanner (Expression 10000XL) (Epson Inc., Nagano, Japan) and analyzed with WinRHIZO/WinFOLIA software (Regent Instruments Inc., Ville de Québec, QC, Canada).

### 4.3. Measurements of REL, Chlorophyll, and Root Activity

Leaves, stems, and roots were collected separately at the end of the treatment period. Their REL was measured as described by Wang et al. [64]. Chlorophyll was extracted with 80% acetone, and the concentrations of chlorophyll a and b were determined with a UV-1800 system (Shimadzu, Kyoto, Japan). To analyze root activity, samples were collected on Days 0, 1, 3, 7, 10, and 15 of stress treatment. The TTC method was applied for qualitatively and quantitatively assessing root activity, which was defined as the capacity for deoxidization (mg/g FW/h) [65].

### 4.4. Determination of MDA and ROS, and Activity of Antioxidant Enzymes

MDA and ROS (H_2_O_2_ and O_2_^−^) levels, as well as the activities of POD, CAT, and SOD, were determined using specific detection kits according to the manufacturer’s instructions (Nanjing Jiancheng Bioengineering Institute, Nanjing, China). For example, MDA was determined using Malondialdehyde (MDA) assay kit (TBA method). H_2_O_2_ was determined with Hydrogen Peroxide assay kit. O_2_^−^ was indicated by anti-O_2_^−^ activity using Inhibition and Produce Superoxide anion assay kit, and the anti-O_2_^−^ activity was negatively correlated with the accumulation of O_2_^−^. POD was determined using Peroxidase assay kit. CAT was determined using Catalase (CAT) assay kit (Visible light). SOD was determined using Total Superoxide Dismutase (T-SOD) assay kit (Hydroxylamine method). Total protein contents of the samples were measured by Coomassie brilliant blue G-250 staining method [66].

### 4.5. Quantification of Free Polyamines

Free polyamines were extracted and derivatized as described by Gong et al. [19,67], with a slight modification, using 1,6-hexanediamine as an internal standard. Briefly, 0.1 g of frozen leaf sample was ground to a fine powder and homogenized with 5% perchloric acid (PCA) buffer. The mixture was incubated on ice for 30 min. After centrifugation, the supernatant was collected and the deposition layer was extracted with 5% PCA buffer again. The crude extracted supernatant was derived with benzoyl chloride at 37 °C for 25 min. The benzoyl-PAs were then leached with ethyl ether and centrifuged at 8000× *g* for 5 min before the upper phase was collected and vacuum-dried in a concentrator (Eppendorf, Hamburg, Germany). The dried extracts were re-dissolved with 300 μL of HPLC-grade methanol (Fisher, Hampton, NH, USA) and filtered. From this, 20-μL aliquots were loaded into an Agilent 1260 Infinite HPLC system (Agilent, Santa Clara, CA, USA) equipped with a C_18_ reversed phase column (4.6 mm × 250 mm; particle size 5 μm) and a diode array detector. The mobile phase comprised HPLC-grade methanol (eluent A) and water (eluent B) and followed a gradient of 65%:35% (*v*/*v*, A:B) to 95%:5% over 15 min, at a flow rate of 1.0 mL/min.

### 4.6. Quantitative PCR Analysis

We used quantitative PCR (qPCR) to analyze the transcript levels of genes involved in the synthesis of PA in alkaline-stressed seedlings. Total RNA was extracted with RNAiso Plus (Takara, Kusatsu, Japan), and 1 μg of the total RNA was used to synthesize the first strand cDNA using PrimeScript™ imeScriptas used to synthesiz (Takara, Japan) according to the instructions. qPCR was performed with a SYBR Premix Ex Taq kit (TaKaRa, Japan) on a Stepone Plus System (ABI, Foster, CA, USA). The PCR solution, in a total volume of 10 μL, contained 5 μL of 2× SYBR Premix Ex Taq (Tli RnaseH Plus), 50 ng of cDNA, and 0.25 μM of each primer (Supplemental Table S1). The reaction conditions were as follows: 95 °C for 30 s, then 40 cycles of 95 °C for 5 s, 56 to 58 °C for 10 s, and 72 °C for 15 s. Each sample was analyzed in four replicates, and the ^∆∆^CT method was applied to calculate relative expression. As an internal control, *Malate dehydrogenase* (*MDH*) (Supplementary Materials, Table S1) was used to normalize the relative expression levels for our genes of interest [68]. 

### 4.7. Statistical Analysis

All data were analyzed with the SAS statistical software package (version 8.1, SAS, Cary, NC, USA). Analysis of variance (ANOVA) was used to compare the results based on Duncan’s multiple range tests. Differences among treatments were considered significant at *p* < 0.05.

## Figures and Tables

**Table 1:** Effects of 5 μM melatonin treatment on growth of *Malus hupehensis* Rehd. seedlings under alkaline stress.

	Shoot Height (M ± SD, cm)			Leaf Number (M ± SD)			Stem Diameter (M ± SD, mm)		
	0 Day	15 Days	Relative Growth	0 Day	15 Days	Relative Growth	0 Day	15 Days	Relative Growth
**C**	10.77 ± 0.23 bc (100%)	13.02 ± 0.28 a (100%)	20.9%	10.67 ± 0.35 c (100%)	13.87 ± 0.19 a (100%)	30.0%	1.88 ± 0.04 b (100%)	2.13 ± 0.04 a (100%)	13.3%
**AL**	10.45 ± 0.30 c (97.0%)	11.57 ± 0.27 b (88.7%)	10.7%	10.67 ± 0.35 c (100%)	12.20 ± 0.24 b (88.0%)	14.3%	1.88 ± 0.03 b (100%)	1.90 ± 0.05 b (87.3%)	1.6%
**C + MT**	10.97 ± 0.48 bc (100%)	13.15 ± 0.32 a (100%)	19.9%	11.33 ± 0.33 c (100%)	13.93 ± 0.21 a (100%)	23.0%	1.90 ± 0.07 b (100%)	2.16 ± 0.04 a (100%)	13.7%
**AL + MT**	10.95 ± 0.39 bc (99.8%)	13.09 ± 0.43 a (99.5%)	19.5%	11.20 ± 0.47 c (98.9%)	13.80 ± 0.17 a (99.1%)	23.2%	1.89 ± 0.04 b (99.5%)	2.11 ± 0.03 a (97.7%)	11.6%

All data are means ± SD of 20 seedlings. The percentages in parentheses after the SD means the data in AL group versus in C group, and in AL + MT group versus in C + MT group. Letters indicate significant difference according to the Duncan’s multiple range test (*p* < 0.05).

**Table 2:** Effects of 5 μM melatonin treatment on tissue fresh weights (FW, g) measured from *Malus hupehensis* Rehd. seedlings under alkaline stress.

	Root (M ± SD)			Stem (M ± SD)			Leaf (M ± SD)			Total (M ± SD)		
	0 Day	15 Days	Relative Growth	0 Day	15 Days	Relative Growth	0 Day	15 Days	Relative Growth	0 Day	15 Days	Relative Growth
**C**	0.5018 ± 0.0248 b (100%)	0.8124 ± 0.03 a (100%)	61.9%	0.2486 ± 0.0097 c (100%)	0.3382 ± 0.0159 ab (100%)	36.0%	0.7468 ± 0.0332 c (100%)	1.2612 ± 0.0523 ab (100%)	68.9%	1.4972 (100%)	2.4118 (100%)	61.1%
**AL**	0.5091 ± 0.0364 b (101.5%)	0.5794 ± 0.0119 b (71.3%)	13.8%	0.2429 ± 0.0145 c (97.7%)	0.2460 ± 0.0127 c (72.7%)	1.3%	0.7401 ± 0.0491 c (99.1%)	0.8650 ± 0.0278 c (68.6%)	16.9%	1.4921 (99.7%)	1.6903 (70.1%)	13.3%
**C + MT**	0.5329 ± 0.0302 b (100%)	0.8571 ± 0.0234 a (100%)	60.8%	0.2581 ± 0.0201 c (100%)	0.3538 ± 0.0124 a (100%)	37.1%	0.7830 ± 0.0470 c (100%)	1.3722 ± 0.0629 a (100%)	75.2%	1.5740 (100%)	2.5831 (100%)	64.1%
**AL + MT**	0.5431 ± 0.0385 b (101.9%)	0.8337 ± 0.0225 a (97.3%)	53.5%	0.2538 ± 0.0182 c (98.3%)	0.3048 ± 0.0043 b (86.2%)	20.1%	0.7939 ± 0.0502 c (101.4%)	1.1581 ± 0.0296 b (84.4%)	45.9%	1.5906 (101.1%)	2.2966 (88.9%)	44.4%

All data are means ± SD of 20 seedlings. The percentages in parentheses after the SD means the data in AL group versus in C group, and in AL + MT group versus in C + MT group. Letters indicate significant difference according to the Duncan’s multiple range test (*p* < 0.05).

**Table 3:** Effects of 5 μM melatonin treatment on tissue dry weights (DW, g) measured from *Malus hupehensis* Rehd. seedlings under alkaline stress.

	Root (M ± SD)			Stem (M ± SD)			Leaf (M ± SD)			Total (M ± SD)		
	0 Day	15 Days	Relative Growth	0 Day	15 Days	Relative Growth	0 Day	15 Days	Relative Growth	0 Day	15 Days	Relative Growth
**C**	0.1175 ± 0.005 b (100%)	0.1801 ± 0.0092 a (100%)	53.3%	0.1042 ± 0.0035 b (100%)	0.1265 ± 0.0067 a (100%)	21.4%	0.2488 ± 0.0118 b (100%)	0.3950 ± 0.0192 a (100%)	58.8%	0.4705 (100%)	0.7017 (100%)	49.1%
**AL**	0.1163 ± 0.007 b (99.0%)	0.1360 ± 0.0043 b (75.5%)	16.9%	0.1036 ± 0.0060 b (99.4%)	0.0989 ± 0.0047 b (78.2%)	−5.5%	0.2404 ± 0.0159 b (96.6%)	0.2792 ± 0.0092 b (70.1%)	16.1%	0.4603 (97.8%)	0.5141 (73.3%)	11.7%
**C + MT**	0.1150 ± 0.007 b (100%)	0.1908 ± 0.0083 a (100%)	65.9%	0.1032 ± 0.0077 b (100%)	0.1427 ± 0.0066 a (100%)	38.3%	0.2544 ± 0.0177 b (100%)	0.3974 ± 0.0109 a (100%)	56.2%	0.4726 (100%)	0.7309 (100%)	54.7%
**AL + MT**	0.1160 ± 0.008 b (100.9%)	0.1796 ± 0.0047 a (94.1%)	54.8%	0.1060 ± 0.0074 b (102.7%)	0.1257 ± 0.0022 a (88.1%)	18.6%	0.2568 ± 0.0204 b (100.9%)	0.3579 ± 0.0070 a (90.1%)	39.4%	0.4787 (101.3%)	0.6631 (90.7%)	38.5%

All data are means ± SD of 20 seedlings. The percentages in parentheses after the SD means the data in AL group versus in C group, and in AL + MT group versus in C + MT group. Letters indicate significant difference according to the Duncan’s multiple range test (*p* < 0.05).

**Table 4:** Effects of 5 μM melatonin treatment on root growth from *Malus hupehensis* Rehd. seedlings under alkaline stress.

	Length (cm)	Surface Area (cm^2^)	Avg. Diam (mm)	Root Volume (cm^3^)	No. of Tips *	No. of Forks *
**C**	244.30 ± 14.93 a (100%)	31.63 ± 2.25 ab (100%)	0.44 ± 0.02 ab (100%)	0.36 ± 0.04 ab (100%)	1577 ± 155 ab (100%)	2275 ± 144 ab (100%)
**AL**	220.46 ± 12.94 a (90.2%)	27.13 ± 1.69 b (85.8%)	0.38 ± 0.01 b (86.4%)	0.29 ± 0.02 b (80.6%)	1179 ± 100 b (74.8%)	2002 ± 140 b (88.0%)
**C + MT**	267.66 ± 21.53 a (100%)	35.75 ± 1.36 a (100%)	0.50 ± 0.02 a (100%)	0.45 ± 0.01 ab (100%)	1944 ± 186 a (100%)	2956 ± 301 a (100%)
**AL + MT**	241.41 ± 20.45 a (90.2%)	31.96 ± 2.21 ab (89.4%)	0.43 ± 0.03 ab (86.0%)	0.46 ± 0.07 a (102.2%)	1822 ± 196 a (93.7%)	2773 ± 253 ab (93.8%)

* The number of tips and forks is a measure of the branchiness of the fine root system, much more number of tips and forks means easier uptake of water and nutrients through the fine roots. All data are means ± SD of 10 seedlings. The percentages in parentheses after the SD means the data in AL group versus in C group, and in AL + MT group versus in C + MT group. Letters indicate significant difference according to the Duncan’s multiple range test (*p* < 0.05).

## Supplementary Materials

The Supplementary Materials are available online. Table S1: Primers used to detect genes involved in polyamine biosynthesis.
