# Supplementary material for: Exogenous Melatonin Alleviates Alkaline Stress in Malus hupehensis Rehd. by Regulating the Biosynthesis of Polyamines
Source: Molecules. 2017 Sep 13;22(9):1542. doi: 10.3390/molecules22091542 (PMC6151414; doi:10.3390/molecules22091542)
Supplement: Supplementary file 1 [file molecules-22-01542-s001.pdf]

Supplemental Table 1. Primers used to detect genes involved in polyamine biosynthesis.

Genome accession No. in GDR	Name	Abbreviation	Primer sequence	Products length (bp)
MDP0000813339	Arginine decarboxylase 1	*ADC1*	F: GATAGCTCTCTTCCCGCGTC<br>R: CGATTCGGTAGAGGTCGGAT	111
MDP0000228682	Arginine decarboxylase 2	*ADC2*	F: AGCTTGGCGTAATCATGGTC;<br>R: ATGCTTCCGGCTCGTATGTT	86
MDP0000914975	Ornithine decarboxylase 1	*ODC1*	F: CAAGTGTACCGTGGAGCAAT;<br>R: TGCAACCATTGTGAAAGCCA	249
MDP0000264258	Ornithine decarboxylase 2	*ODC2*	F: GGTTGAGAGTGGTGGGAGTT;<br>R: CAACCAAAGTGAAAGGCGT	300
MDP0000171041	*S*-adenosylmethionine decarboxylase 1	*SAMDC1*	F: CGGTCACGTACCGCA;<br>R: TCATGCATCCAGGAAGTCTC	292
MDP0000757066	*S*-adenosylmethionine decarboxylase 2	*SAMDC2*	F: CTGGTTCGCAGTCTCCAAGG;<br>R: AAACAGCAGCGACACAGCAA	221
MDP0000292444	*S*-adenosylmethionine decarboxylase 3	*SAMDC3*	F: CATTCTACGAAGCTCCCCTC;<br>R: GACCGGTACAGCCATGTTAG	104
MDP0000211726	*S*-adenosylmethionine decarboxylase 4	*SAMDC4*	F: CGGTCCTCTGAAGAGAGTGC;<br>R: GGTCGGATGTCTTCGATGCT	155
MDP0000120546	*S*-adenosylmethionine decarboxylase 5	*SAMDC5*	F: TCAACTCTCAAGGGCTGCTG;<br>R: GCTGCATCACGAGAAACGAC	206
MDP0000198590	Spermidine synthase 1	*SPDS1*	F: CGAGCGCCTTTTTATCCCGT;<br>R: GGAGGCAAAAACTAGCTGGC	208

MDP0000027925	Spermidine synthase 2	*SPDS2*	F: TGGTCCCTGTCTCAGAGTGT;<br>R: GCCAAACGCACCAAAGGAAT	112
MDP0000521365	Spermidine synthase 3	*SPDS3*	F: CCCTATGTGGCCAGTCCTCAAT;<br>R: GATGGCGAGAAACCTCCCT	192
MDP0000294813	Spermidine synthase 4	*SPDS4*	F: CAACCCAGGCAAGATCAACC;<br>R: TTCTCCGTCTCCAGTCTAGC	107
MDP0000788247	Spermidine synthase 5	*SPDS5*	F: TCGCCATCCTTCTGTTGAGC;<br>R: CAACAGCCAACCGAGGAAAAA	92
MDP0000162897	Spermidine synthase 6	*SPDS6*	F: GTTTCTCGCCATCCTTCTGTT<br>R: CTTCCCTTTAGGTGCAAGCCT	171
	Malate dehydrogenase	*MDH*	F: CGTGATTGGGTACTTGGAAC<br>R: TGGCAAGTGACTGGGAATGA	113

*PA-related genes were identified from GDR database (https://www.rosaceae.org/) and designated according to their homologous in *Arabidopsis*. The primers of *MDH* was described by Perini et al. [1].

1. Perini, P.; Pasquali, G.; Margis-Pinheiro, M.; de Oliviera, P.R.; Revers, L. Reference genes for transcriptional analysis of flowering and fruit ripening stages in apple (*Malus* x *domestica* borkh.). *Mol Breeding* **2014**, *34*, 829-842.
